# Inferring molecular inhibition potency with AlphaFold predicted structures

**DOI:** 10.1038/s41598-024-58394-z

**Published:** 2024-04-08

**Authors:** Pedro F. Oliveira, Rita C. Guedes, Andre O. Falcao

**Affiliations:** 1 Lasige, Faculdade de Ciências, Universidade de Lisboa, 1749-016 Lisboa, Portugal; 2 Research Institute for Medicines (iMed.ULisboa), Faculdade de Farmácia, Universidade de Lisboa, Av. Prof. Gama Pinto, 1649-003 Lisboa, Portugal; 3 Departamento de Informática, Faculdade de Ciências, Universidade de Lisboa, 1749-016 Lisboa, Portugal

**Keywords:** In silico drug discovery, Quantitative structure-activity relationship modeling (QSAR), Structure based virtual screening, Machine learning, Protein structure, Proteo-chemometrics, Computational models, Virtual screening

## Abstract

Even though in silico drug ligand-based methods have been successful in predicting interactions with known target proteins, they struggle with new, unassessed targets. To address this challenge, we propose an approach that integrates structural data from AlphaFold 2 predicted protein structures into machine learning models. Our method extracts 3D structural protein fingerprints and combines them with ligand structural data to train a single machine learning model. This model captures the relationship between ligand properties and the unique structural features of various target proteins, enabling predictions for never before tested molecules and protein targets. To assess our model, we used a dataset of 144 Human G-protein Coupled Receptors (GPCRs) with over 140,000 measured inhibition constants (K_i_) values. Results strongly suggest that our approach performs as well as state-of-the-art ligand-based methods. In a second modeling approach that used 129 targets for training and a separate test set of 15 different protein targets, our model correctly predicted interactions for 73% of targets, with explained variances exceeding 0.50 in 22% of cases. Our findings further verified that the usage of experimentally determined protein structures produced models that were statistically indistinct from the Alphafold synthetic structures. This study presents a proteo-chemometric drug screening approach that uses a simple and scalable method for extracting protein structural information for usage in machine learning models capable of predicting protein-molecule interactions even for orphan targets.

## Introduction

In the field of drug discovery, *in silico* approaches have been actively explored in numerous studies because of their potential to accelerate the full process and offer cost-effectiveness, as computer models should be able to pre-screen huge molecular databases, selecting the most promising active compounds. Many new drug compounds have been successfully developed with the aid of computational methods^1,2^. Currently, drug discovery pipelines take advantage of several *in silico* methods used in drug discovery. Some of these methods include computational identification of potential drug targets, virtual screening of large chemical libraries for effective drug candidates, optimization of hit compounds, and *in silico* assessment of their potential toxicity.

Approaches for in silico drug discovery can be broadly divided into ligand-based drug discovery (LBDD) and structure-based drug discovery (SBDD)^1,3,4^. SBDD techniques use both molecular and target structures to try to predict how molecules might bind and try to find molecules with high affinity, aiming to understand how drugs interact with proteins and to create more effective and specific medications. SBDD methods include some popular methods such as molecular docking and structure-based virtual screening^1,5–8^.

**Figure 1:**
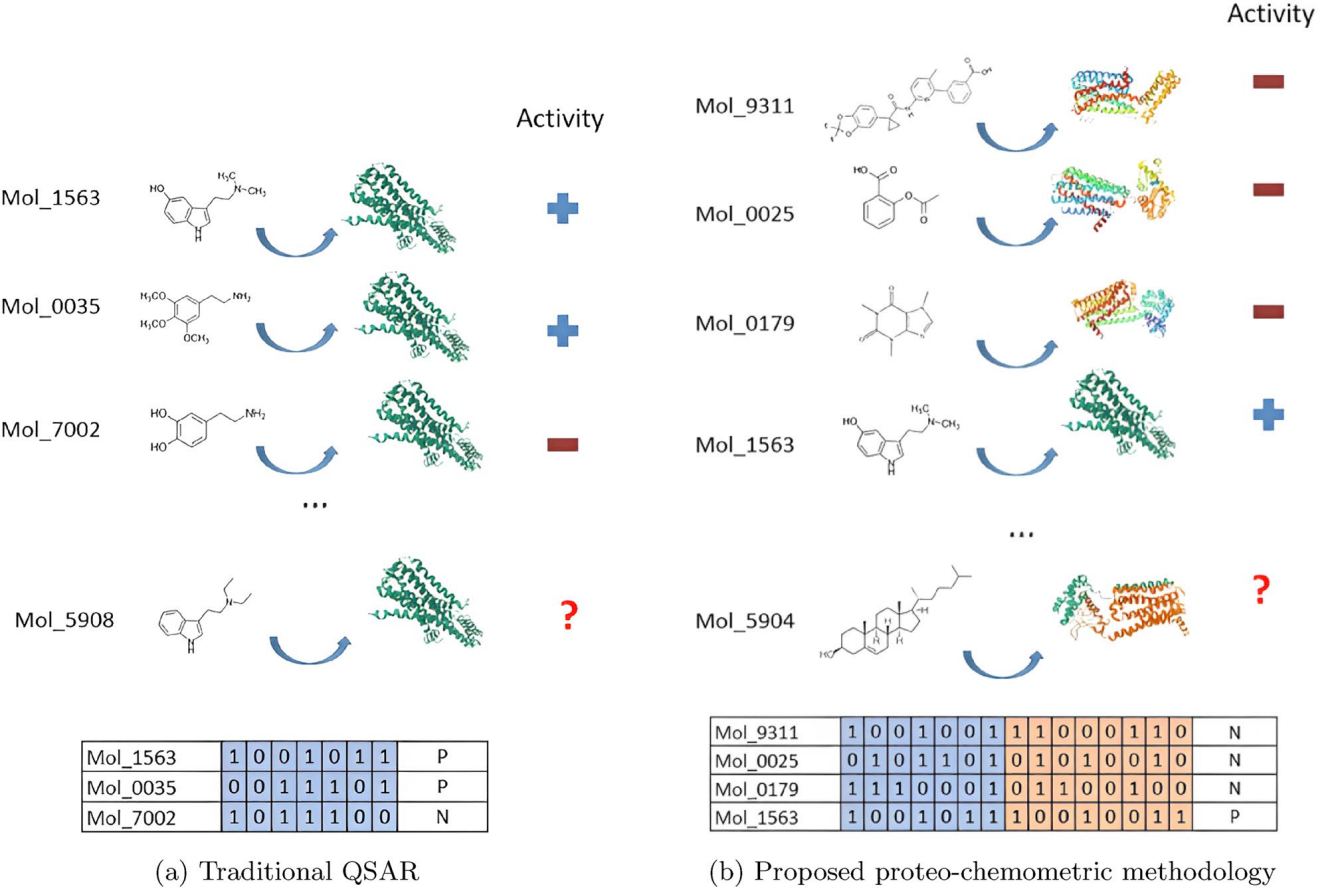
Visual representation of the traditional QSAR approach (**a**) where the descriptors of the molecules with known activities are used to create a model capable of predicting activities for untested molecules. On the left (**b**), is a visual representation of the proposed proteo-chemometric methodology where fingerprints for multiple targets and molecules are used to create the model.

On the other hand, LBDD generally uses only the chemical and structural characteristics of the ligands themselves and is used to identify and design new drugs based on the properties of known ligands or molecules for one specific target and, as such, requires some previous experimental results of binding activity, to create models that should be able to identify other molecules with similar activity profiles. The use of Quantitative Structure-Activity Relationship (QSAR) models is one of the most used LBDD approaches, using generally a combination of state-of-the-art machine learning models and different representations of molecules^9,10^. QSAR models, as most Machine Learning models, perform better when the dependent variable is the same for all observations. Therefore, for the large majority of QSAR studies, modelers select one specific target for which there is already some quantitative data with actual observations and try to identify the characteristics of the molecules that potentiate similar behavior to the active ones in the data set (Fig. 1a). Depending on the quality and quantity of available data, it is nonetheless possible to develop robust and effective predictive models for individual targets for which there exists reliable molecular activity data.

By integrating QSAR models and machine learning into the drug discovery pipeline, researchers can efficiently screen large chemical libraries, prioritize compounds for synthesis and testing, optimize lead compounds, and gain insights into the underlying structure-activity relationships^11^. This approach has been shown to accelerate the drug discovery process, reduce costs, and aid in the identification of novel drug candidates with improved potency, selectivity, and safety profiles^12^.

One of the fundamental shortcomings of QSAR modeling is that models must be constructed from available data. For a target that has never been targeted in *in vitro* measurements, it is not generally possible to create any type of inference model. Some approaches have emerged that included target-based information into models like protein sequence similarity^13^ or amino acid properties of ligand sites^14^, yet all of these approaches either do not use the full protein structural information, or they require extensive knowledge of the protein structures, which may limit their applicability in large-scale analysis. The field of proteo-chemometrics^15,16^ methods aim to address these problems by making models using several targets with information from both targets and ligands, and therefore our approach can be classified among these methods.

The goal of this work is to present a different proteo-chemometric approach for modeling target based data, focusing on structural neighborhoods, so that the generated model may identify the specific target characteristics to which ligands may bind to. Following the main insights of proteo-chemometrics, it is aimed to produce a single model that should be able to make predictions for any combination of ligand and protein target. A successful model should be able to make predictions for orphan targets or targets that were never even assessed or even isolated in *in vitro* essays. To accomplish this goal, it is required to have complete and sufficiently reliable protein structural information that can be used for modeling. To address this, the PDB database would be an obvious choice, however the inconsistency and incompleteness of many entries^17,18^, with a significant percentage of these files having missing residues and incomplete protein sections^19,20^ makes such usage difficult for many targets. Because of that, numerous software programs have been developed to address these deficiencies^21–23^. This, coupled with the large number of structure determination methods, and a nonexistent standard structure for each protein, makes the usage of PDB data complex and error-prone. As a possible solution, and built from all the structural data available, AlphaFold, an Artificial Intelligence (AI) system, has demonstrated remarkable accuracy in predicting protein folding^24^. With the availability of over 200 million structures in the AlphaFold Database^25^, researchers now have the opportunity to work with the structures of virtually any protein in existence. Therefore an obvious and simple solution for getting protein structures for modeling problem would be the usage of AlphaFold generated data, for which there are generated structures for most proteins catalogued in databases, and, despite the known flaws^26,27^, would provide us general predicted structure data, that could be used for very large modeling efforts potentially encompassing full databases with millions of ligand-target biding activities. Further, and of special relevance to our work, it has been demonstrated that Alphafold structures have been used to enhance predictions of binding affinities for GPCRs^28^. This same idea has been proposed in other studies(e.g.^29^) with a very different approach for encoding proteins and molecules (e.g.^15,30^) which use distinct approaches like ligand and target similarity, or encoding of protein amino-acid sequences, differing widely from the methods proposed in this study, as well as in the global objectives.

In summary this study has two general goals: A) to demonstrate that a method capable of the encoding 3-Dimensional structures of proteins is capable of producing machine learning models that encompass both Target and Ligand information. This will be made possible by the wide availability of Alphafold predicted protein structures, as well as a much larger data set of binding activities provided by the ChEMBL database^31^; and B) we aim to demonstrate that this approach is capable of making global models that are able to predict binding affinities for any target, even if no binding information is available. This will be accomplished by a very strict validation procedure, that cannot be measured through commonly available benchmarks (e.g.^32,33^), in which all the binding information for a set of targets will be totally hidden from the model during the training phase, so the model will have to make predictions for targets never used before.

It is important to state from the beginning that the goal of this study is not to find the best machine learning algorithm to solve these types of problems, but rather to identify whether or not this modeling approach is capable of showing promise and finding definite signal in making predictions. The focus is not on searching for the best descriptor set for molecules, or even the best possible Machine Learning modelling approach, but rather to verify if the highly scalable approach developed, made possible by the wide availability of good structural data for virtually every protein in existence as provided by AlphaFold, is capable of making reliable inference even for targets without any structure measured. The authors further believe that no existing benchmark is extant that actually aims for validation with binding information for targets not present on the training set, and this is one of the critical goals of this study.

## Methods

As stated above, the first fundamental idea for this study is to describe how to build a single Machine Learning model that includes both the ligand and the target structural data. The resulting model should be able to make inference both for unknown ligands and proteins (Fig. 1b). As such, after a set of proteins with available ligand activity data has been selected, the challenge is to select a way to encode the structures of the ligands and targets in a way that they could be used for fitting a Machine Learning model.

### Structural information of small molecules

The usage of molecular fingerprints has proven to be one of the most reliable methods for capturing structural information of small molecules, and has been used with success in many QSAR studies^34–36^. The concept of molecular fingerprints is founded on the idea that the molecules structural and chemical characteristics can be encoded into a binary string, each bit corresponding to the presence or absence of a specific structural characteristic^37^. Several reviews and comparative studies are extant, comparing the suitability of different approaches (e.g.^37–39^) and more sophisticated approaches using probabilistic methods^40^ as well as promising new results^41^ are emerging from using Shannon Entropy descriptors^42^. However, the Extended- Connectivity Fingerprints (ECFPs)^43^ based on Morgan’s algorithm^44^ have been consistently among the most common and reliable approaches and have been tested and used in several modeling studies (e.g.^45,46^). In ECFPs, each atom is extended through its covalent connections up to a predefined radius, defining a unique substructure in the molecule. Each of those substructures is then encoded as a fixed structure, then for each circular layer, paths are generated by considering all possible combinations of bonds and atom types within the radius. These paths are encoded and then hashed to create unique identifiers for the local chemical substructures (fragments) around each atom. Finally, each unique substructure obtained from the path encoding step is hashed into a fixed-length binary bit string. Each bit in the fingerprint corresponds to the presence (1) or absence (0) of a specific substructure in the molecule^43^.

### Encoding protein structural features

Using structural features of small molecules data is a fairly common procedure in QSAR modeling, however it is still a challenge to incorporate structural features of proteins, for which even though there are several approaches, they differ in goal, method and simplicity of application^15,16,29,30,47^. Thus, as referred, one of the primary goals of this study is to demonstrate a highly scalable and simple approach for incorporating proteins’ structural information into QSAR modeling to enable predictions for multiple targets. The usage of fingerprinting for describing protein structures is not new, for instance the molecular surface interaction fingerprinting^47^. Yet these are generated by deep-learning networks with the purpose of predicting protein pocket-ligand and protein–protein interaction site predictions.

The approach followed here is actually of direct application to protein structures, with no learning procedure involved and is highly scalable and of fast application for any protein. The essential idea is actually similar to the above described Morgan fingerprints, however instead of following a covalent graph distance as in ECFP, for each atom in the protein, its 3D neighborhood is examined, checking the presence of amino acids within a given radius from that atom. An amino acid is considered as part of a neighborhood if any of its atoms fall within the specified radius of that central atom. After sweeping all the atoms of the protein, duplicate entries are removed since certain atoms may share exactly the same neighbors set. This process results in a collection of proximal regions composed of protein residues for each protein. Each of those regions is then encoded as a string, which is sorted and then hashed into an integer. This value corresponds to an index that will be set to 1 in the resulting protein fingerprint. By using hash maps, it is possible that two different structures are hashed into the same index, thus highlighting the same bit. This is a *hash collision* and is assumed to occur in fingerprints, eventually becoming a source of errors. Depending on the bit size of the fingerprint array, this may not be very common, with the number of collisions decreasing as the number of bits considered increases.

**Figure 2:**
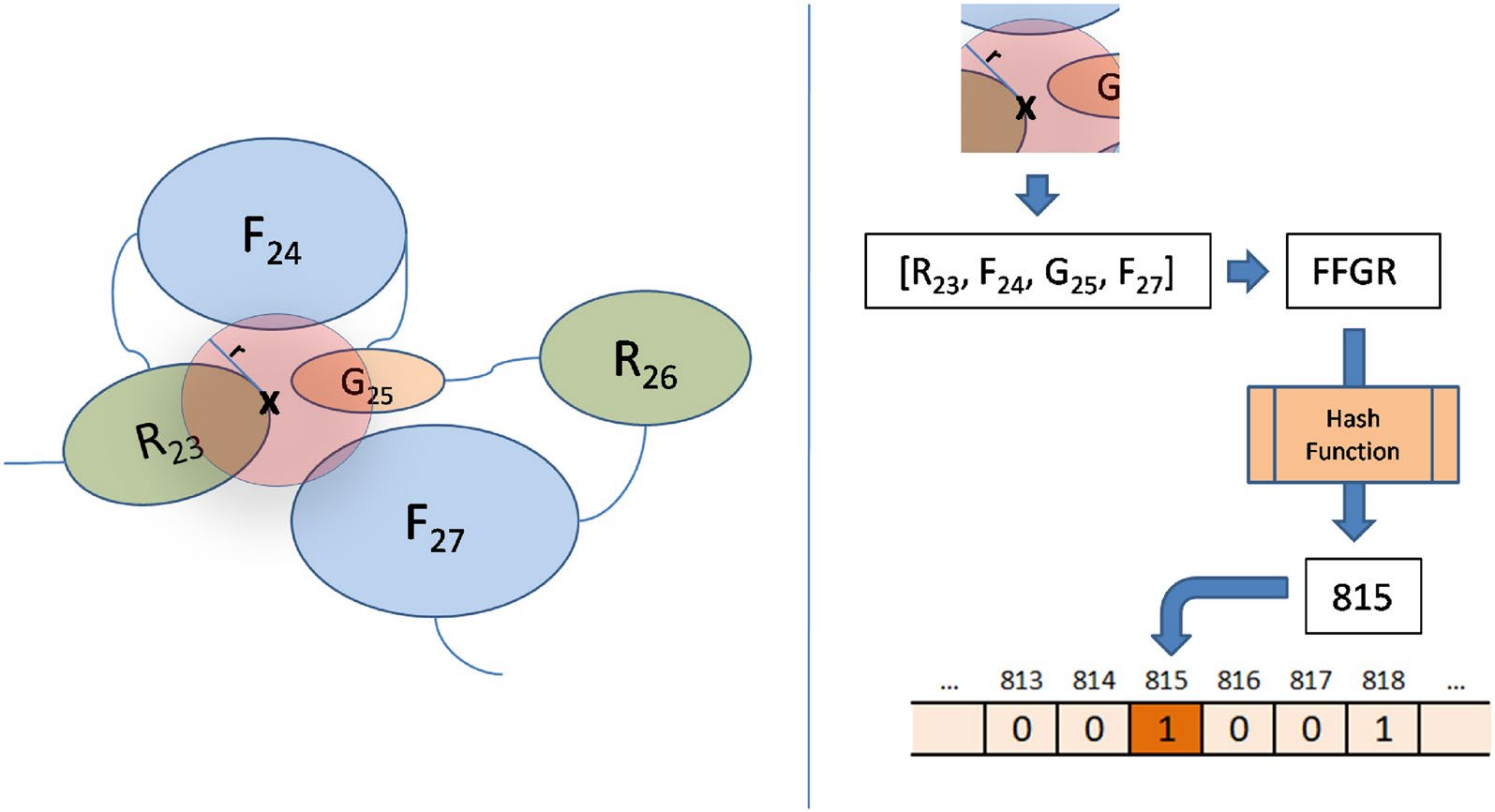
Generating proteins structural fingerprints from close amino acids.

In the example (Fig. 2) *X* represents an atom from Arginine at position 27 (coded as $R_{27}$) of the protein sequence. Within a specified radius *r*, atom *X* is close to amino acids $F_{24}$, $G_{25}$ and $F_{27}$. Therefore, its neighborhood is defined as $R_{23}F_{24}G_{25}F_{27}$. Although two phenylalanines ($F_{24} and F_{27}$) are present, they appear in different positions in the protein sequence, so both will be included in the neighborhood structure, which is finally sorted into a final list of close amino acids encoded as as [*FFGR*]. This is coded into an integer (815, in this example) that will set its corresponding bit as one in the protein structure fingerprint array.

In summary, the method involves defining a radius, identifying amino acids within that radius, representing them as sets of different amino acids, and finally encoding it them into a binary vector using a hash function. The resulting binary vector whose size is defined beforehand is a representation of all residue topological patterns in a single protein.

### Fitting, evaluating and validating models

Machine learning algorithms are widely employed to develop QSAR models due to their ability to recognize complex structural patterns related to the binding activity of a set of ligands to a given target. These algorithms can be trained on diverse chemical datasets to learn the relationships between molecular features and activity. Popular machine learning methods in QSAR include random forests^48^, support vector machines (SVM)^49^ , artificial neural networks (ANN), and other ensemble methods,^50,51^. Like most supervised learning models, they require a thorough validation procedure, which is critical for their applicability beyond the boundaries of the dataset^35,52^.

**Figure 3:**
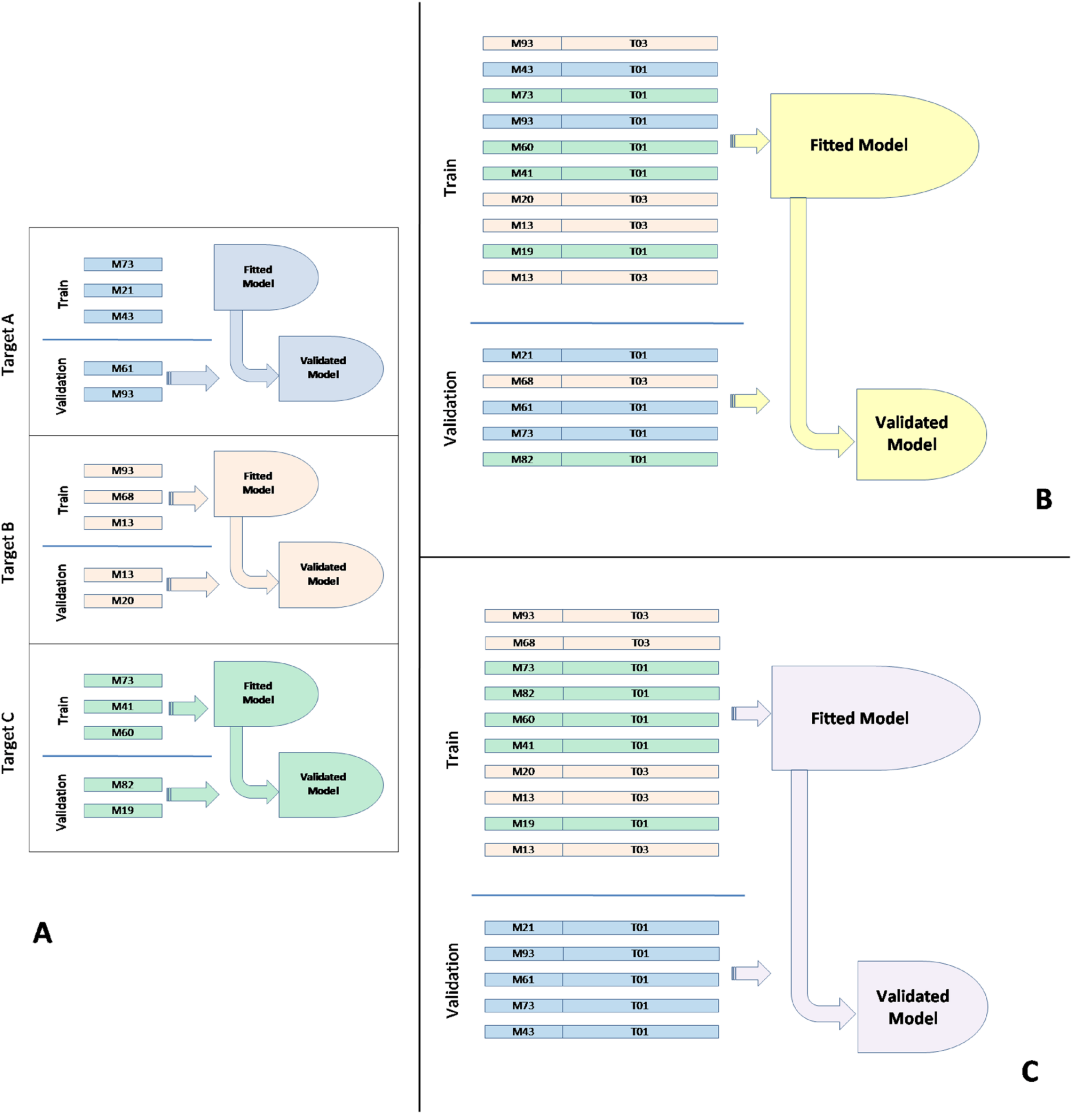
The three modeling approaches followed. (**A**) Baseline individual QSAR models without any target based information; (**B**) Unified model with both ligand and target structure data with randomly selected data from all targets for validation; (**C**) Unified Blind model built with data from a subset of targets using data from an unknown target for validation.

To effectively evaluate the proposed method, a three-step approach is necessary. (Fig. 3). In the first place, it is required to have a control baseline to which the produced results can be compared. As such, a set of classical single-target-QSAR modeling approaches is to be employed first, fitting current state-of-art models with separate data sets for each target. During this phase, data for each target will be split between training and validation sets, where a separated validation set is to be used once for assessing the quality of the produced models^52^. (Fig. 3A). In this case, as in the others, it is important to clarify in Fig. 3 that the *Fitted Models* presented are not changed in any way by the validation set to produce *Validated Models*, but the models are validated in the sense that we used external and independent data sets to evaluate them and produce reliable statistics for each.

Having defined the baseline approach, the first core method of this work involves creating a *Unified Model* coupling all structural data for all molecules and target proteins in the data set, as described above. The fundamental idea is to generate a single model that should be able to make predictions for different combinations of ligands and target proteins. For this approach, a random separation of data between training and validation sets is performed, meaning that all targets could potentially appear both in the training and validation sets (Fig. 3B). Once again, the validation set is used only once for assessing the quality of the produced model. This approach would allow to assess how well the target structural data is able to subsume the requirement for individual models.

The Unified Model, as defined above, although even if yielding positive results, does not allow us to address the primary problem we are a trying to address, which is making *ab initio* predictions for targets never before assessed, as data from all targets may appear scattered through both the training and validation sets. As such, a third modeling approach was devised, where a single model is fitted using data resulting from a subset of all targets, and the produced model is validated with the data from the left over targets (Fig. 3C). If this latter approach proves successful, it indicates the possibility of using structural data from both ligands and protein targets for general inference, even for proteins that were never assessed for binding affinity or even isolated in *in vitro* studies. This, expectedly much more challenging approach, creates a *Blind Model*, as the generated model will be tested on data from targets that it has never “observed” before.

#### Model validation

The general fitting and validation procedure procedure starts with a data set split where each for each model’s data is randomly divided into training and validation sets, with the training set comprising 80% of the data. For model quality assessment, a 5-fold cross-validation it applied to the training set, for eventually identifying the optimal hyper-parameters for the model fitting approach. The evaluation metrics used were the Root Mean Squared Error (RMSE) and Ratio of Explained Variance (RVE). Finally, when the resulting model is selected and evaluated, it is then validated on the validation set, which is used only once, and the results are recorded. This was the approach followed for the generation of Baseline Models and the Unified Model.

For the Blind Model, a different approach was followed for train validation split, where a set of targets is selected, randomly or according to a specific criterion, and all their their binding affinities are removed from the main data set, which will be used for training. The removed set of target data will become the validation data.

## Data processing

The data collected for this study can be divided into two main categories: compound bioactivity and protein structures. To retrieve the compound bioactivity data, two resources, UniProt,^53^ and ChEMBL^31^, were used. A specific family of proteins was chosen, namely G-protein-coupled receptors (GPCRs), which are widely recognized as successful therapeutic targets for various diseases^54,55^. The UniProt website was employed to search for the desired proteins and obtain their corresponding identifiers (IDs). The search was filtered to include only human proteins that had undergone human curation (SwissProt). These protein IDs were then utilized to search the ChEMBL database and retrieve the related target IDsand corresponding biological activities. In this process, we selected the inhibition constant (*K*i) bioactivity, as it is more precise than IC50 and its value is independent of the concentration of the substrate.

### Data retrieval, processing and curation

As referred, all molecular structures were retrieved from ChEMBL. Even though this repository is standardized and manually curated, incomplete, contradictory, or uncertain data may occasionally arise. To ensure comparability across different essays that encompass different targets, it is necessary to address these issues with a common set of rules. As such, the following procedures were followed for all situations:

1. Activity values with incorrect units for the given activity type were excluded. All other activity values were converted to nanomolar (nM), which was the predominant unit type.
2. Activity values with an activity relation of “$=$” and no corresponding value, or lacking an activity relation and activity comment, or an activity comment indicating activity without a specified value were excluded. Only active compounds with actual measurements were included in the analysis.
3. If there were two or more measurements for the same target and compound, their difference was checked, and if it exceeded one order of magnitude, the most recent measurement was selected; otherwise, the average of the logarithm of the measurements was calculated and used.
4. If there were one or more activities with reported values, but at least one activity had a sign of “<” or “$\le$” in the activity relation (indicating activity below a certain threshold, but the exact value is unknown), this information was saved as observations. In the case of multiple activities with “<” or “$\le$” relations, the report with the highest value was selected .
5. If there were no defined activities (with an '=' type relation) but one or more activities for the same compound and target reported with “<” or “$\le$” in the activity relation, the transformed value would be calculated for the activity with the highest concentration.
6. No distinction was made for activities with an activity relation of “>” or “$\ge$” (as these generally do not imply activity, but rather inactivity) and therefore the molecule was assigned as not active for that target.

After making the aforementioned data standardization, the *log*(*K*_*i*_) values were further transformed to ensure that they fell within the range of 0 to 1, in a score denominated *spK*_*i*_ for *scaled pK*_*i*_^35,45^. This score considers all *K*_*i*_ values below 1.0 nM as very active, therefore with a score of 1.0, *K*_*i*_ a concentration above 10,000 nM as a non-active, therefore having a score of 0.0, with all other cases receiving a value from a linear interpolation of the *log*(*K*_*i*_) (Equation 1).

1 $$ spK_{i} = \left\{ \begin{array}{*{20}l} 0 &{} {\text {if K}_{i}} \ge 10,000 \text { nM or inactive},\\ 1 & {} {\text {if K}_{i}} \le \ 1.0 \text { nM}, \\ \frac{4-\log _{10}(K_{i})}{4} &{} \text {otherwise} \end{array}\right.  $$

This representation is readily understandable, making easier the interpretation of results. Any molecule active wih *K*_*i*_ values below 1, appear with the highest score, and no difference is made for even lower concentrations, as these are exceedingly active molecules On the other hand no difference whatsoever is made between molecules with *K*_*i*_ values below 10,000 or inactive, as these typically have no physiologically measured effect. Scaling all other values between 0 and 1 further allow us to readily understand the activity profile of any given ligand-target interaction value from inactive to extremely potent in a linear scale.

The final data sets were constructed based on the available information on inhibitory constants for each pair of small molecule - protein target. In total, about 197 different Human GPCRs with available *K*_*i*_ activities were identified. Out of these, 50 had less than 40 *K*_*i*_ values and thus were discarded, and finally, 3 did not show sufficient variability in *K*_*i*_ values, and were eliminated as well. This amounted to 144 targets with 141,225 different activities for a total of 69,879 distinct molecules.

### Data transformation

For generating the molecular fingerprints, the RDKit toolkit, an open-source cheminformatics library^56^, was employed to all structures retrieved from ChEMBL. RDKit provided the required functionality for generating Morgan Fingerprints from the molecular structures, using a molecular radius of 3, and a number of bits of 2048, which is commonly denominated ECFP6, for Extended Connectivity FingerPrints with radius equal to 3.

Regarding the structural information of proteins, the structure files were obtained from the AlphaFold Database^25^, and the process described previously was followed to derive the corresponding protein fingerprints from the protein structures. The source code for generating the protein fingerprints is made available as Supplementary Material. A 5.0Å distance was selected for atom radius for building the protein fingerprints. Each amino acid unique pattern was hashed into a 16,381 bit array (the first prime number below $2^{14}=16,384$).

The much larger array size required for protein fingerprints dwarfed the 2048 bits used for molecular structures. This in itself might not be a problem, however the same exact fingerprint patterns are going to be present for all molecules having any binding activity recorded for each specific target. As only a limited number of targets was tested, this redundancy could significantly hinder model learning. To address this issue, it was decided to use a dimensional reduction approach that could encompass all the protein structural information in the data set albeit with a much smaller number of columns. The approach followed was the standard Principal Component Analysis (PCA), which was exclusively applied to the protein fingerprints. The fingerprints for small molecules were used directly as they appeared in the resulting data sets. The number of components selected should either match the total number number of existing targets or capture at least 99% of the data variance.

### Model fitting

The modeling effort was completed in Python, primarily using the scikit-learn machine learning libraries^57^. An initial model pre-screening phase was used where different types of machine learning approaches were tested for all models. The variety of models used were Random Forests^58^, Support Vector Machines for Regression^59^ and Gradient Boosting Methods^60^. In this preliminary screening, Random Forests with default parameters appear to be very robust producing in general the best results, and were selected for all modeling approaches reported. The only parameter that was changed from the default was the number of trees, which was increased to 200.

## Results

As described above, three different approaches were followed for the 144 targets selected. Firstly, a baseline pure QSAR model was developed, where each model was individually trained and validated for each target using only molecular structural data. Secondly, a unified model that includes both ligand and protein data, where the training and the validation sets containing data from all protein targets. Lastly, a blind inference model was trained using data from a set of targets, with the validation set including only targets that were not on the training set, randomly selected. This latter approach was supplemented with one further refinement, a *Semi-Blind* model where the targets used for validation were selected as being similar to others in the training set. To accomplish this, from the 144 protein set, the Jaccard similarity, using the computed structural protein fingerprints, was computed between all proteins. Then the 15 closest pairs were identified and, for each pair, one protein was selected, including all its measured binding activities to form the validation set (Table 1). For both the Blind and Semi-Blind models, no information about any elements of the validation set was present in the training set.

**Table 1:** List of testing targets for the semi-blind modelling approach and respective similar targets on the training set.

Target GeneID	Name	N. Activities	Similar target on training set	Similarity
HCRTR1	Orexin receptor type 1	1574	Orexin receptor type 2	0.110
CHRM4	Muscarinic acetylcholine receptor M4	853	Muscarinic acetylcholine receptor M2	0.159
CHRM5	Muscarinic acetylcholine receptor M5	780	Muscarinic acetylcholine receptor M1	0.115
ADRB3	Beta-3 adrenergic receptor	335	Beta-1 adrenergic receptor	0.087
ADRA2B	Alpha-2B adrenergic receptor	558	Alpha-2A adrenergic receptor	0.105
ADRA2C	Alpha-2C adrenergic receptor	683	Alpha-2A adrenergic receptor	0.116
DRD5	D(1B) dopamine receptor	526	D(1A) dopamine receptor	0.136
CXCR1	C-X-C chemokine receptor type 1	102	C-X-C chemokine receptor type 2	0.186
HTR1B	Serotonine receptor 1B	1040	Serotonine receptor 1D	0.096
HTR2C	Serotonine receptor 2C	2633	Serotonine receptor 2A	0.094
SSTR1	Somatostatin receptor type 1	185	Somatostatin receptor type 4	0.083
CCR1	C-C chemokine receptor type 1	160	C-C chemokine receptor type 3	0.096
DRD3	D(3) dopamine receptor	5106	D(2) dopamine receptor	0.098
OPRK1	Kappa-type opioid receptor	3907	Delta-type opioid receptor	0.090
CCR5	C-C chemokine receptor type 5	160	C-C chemokine receptor type 2	0.116

For the Baseline QSAR models, the final model statistics (Table 2) were computed as a weighted average of the individual model statistics. On the other hand, for the unified models, since the proportion of ligands for different targets might vary significantly between training and test sets, an extended cross-validation procedure was adopted, as the same general model architecture selected, was fitted to different data partitions and validated separately. The process was repeated 10 times, and the results represent an average of all results. In the blind and semi-blind approaches, this would naturally not be an issue, and the validation was conducted using all the bioactivities from targets that were not used for training.

Aggregated results of the four approaches are presented in Table 2. Full results can be found in the Supplementary Information. As expected, the baseline models, having the advantage of being fit with direct knowledge of each protein target, generally exhibit slightly better performance. On the other hand, the unified model, which does not require separate models, show impressive precision in its inference results, averaging just slightly below the QSAR models. This in itself reveals that the approach followed and the protein structure representation used was capable of identifying structural relationships between ligands and targets, strongly suggesting that the proposed approach can effectively capture protein-ligand relationships and yield results comparable to those obtained using traditional QSAR approaches.

**Table 2:** Results of all modeling approaches - RMSE - corresponds to the Root Mean Squared Errors of all predictions; RVE is the weighted average of the Ratio of the Variance Explained.

Model	Training set size	Testing set size	RMSE	RVE
Baseline QSAR	112,217	28,122	0.167	0.683
Unified model	112,271	28,068	0.167	0.677
Blind model	132,854	7,485	NA	NA
Semi-blind model	121,737	18,602	0.301	0.208

**Figure 4:**
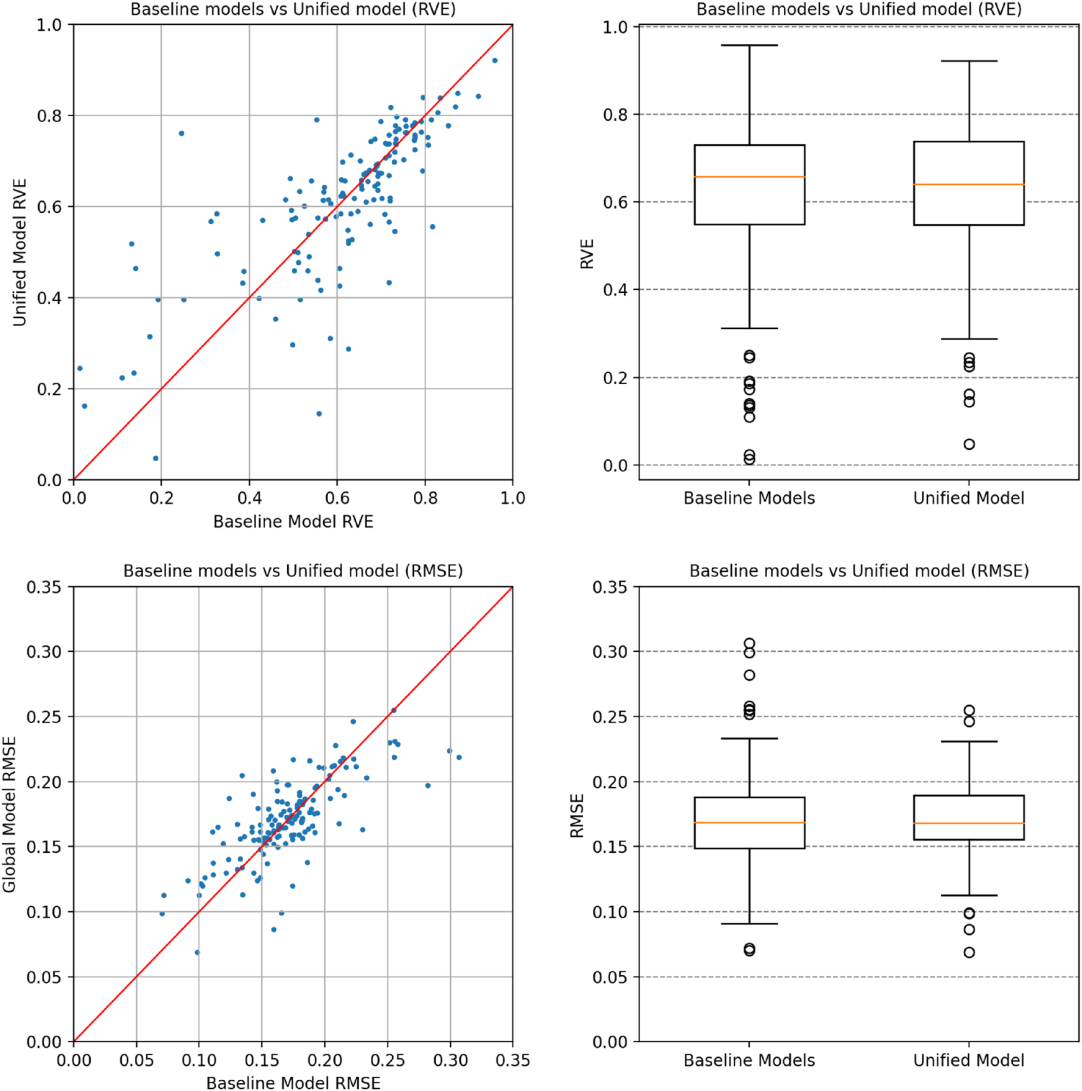
Comparing validation results of Baseline QSAR models and Unified Model for Root Mean Squared Error (RMSE) and Ratio of Variance Explained (RVE) for 144 targets.

It is possible to have a more encompassing view of of how the baseline models compare to the unified model in Fig. 4, where the results for all 144 targets are presented. The scatter plots represent the model results for RVE and RMSE for all 144 targets tested on the Baseline models versus the Unified model. It is possible to verify that in general difficult models for QSAR show similar behavior when compared to the unified model. Yet, there are several models that appear significantly to the left of the 45^∘^ line. For these targets, there is an obvious benefit in the usage of the Unified approach, where the protein structural data coupled with a much larger training data set have an expected quality better than the counterpart QSAR models. This suggests that the larger database used to make the models and the inclusion of structural information for these targets positively impacted the prediction of *pK*_*i*_. The box plots in the same figure illustrate the general behavior of the models for each of the individual protein target. For the RVE, the QSAR-based approach reached a median of 0.658, just slightly above 0.641 for the Unified model. Additionally, it can further be verified that there are much more low-performance outliers in the baseline models, evident in the large trail of target predictions with very low RVE and consequently high RMSE values, reinforcing the idea that the information contained in the protein structure representation is capable of aiding in the prediction results of difficult targets to model.

The Blind and Semi-Blind inference models, in a way, aim to understand whether it is possible to extend the inference to targets never addressed in any models. After extensive testing it was observed that, with a validation data set created from a random partition of target data, we could not identify any structural patterns able to make predictions. Even though all proteins come from the same family with known common structural patterns (GPCRs) we could not identify any signal from data and validation results were indistinct from random predictions.

For the case of the Semi-Blind inference, model results are in general positive. For the large majority of targets it was possible to detect clear patterns that resulted in effective prediction signal with RVE values above 0.2. Such a score may appear as a low threshold, but this was to be expected as the problem is very hard. It is not uncommon, even in QSAR studies, to reach such low prediction values, even in the 144 baseline QSAR models in this study, for about 6 targets, RVE values were below 0.2. - of those, two were actually selected for semi-blind testing, namely CCR5 and CCR1 - On this aspect it is crucial to bear in mind that, when using the Blind and Semi-Blind approaches, we are essentially predicting bioactivities for targets for which we lack any prior binding affinity data, as no information whatsoever about them is present in the training set. Consequently, it is natural and expected for the results to be less favorable compared to those obtained using the previous approaches. Nonetheless, for the Semi-Blind model it is evident that the model is still capable of capturing a part of the relationships between the structural features and the measured spKis. This suggests the potential usefulness of this approach even for *ab initio* predictions, when data for a target was not available. In Table 3, we present the results achieved for each individual target for all 3 approaches (Baseline, Unified and Semi-Blind), and it is worth noting that some cases exhibit particularly promising outcomes. Noteworthy results were obtained for CHRM4, SSTR1, and HTR1B, with an RVE exceeding 0.5.

**Table 3:** Comparison of results for all 15 validation targets of the semi-blind model.

Gene	Baseline models		Unified model		Semi-blind model	
	RMSE	RVE	RMSE	RVE	RMSE	RVE
HCRTR1	0.163	0.660	0.204	0.493	0.288	0.059
CHRM4	0.147	0.770	0.169	0.735	0.221	0.576
CHRM5	0.159	0.617	0.168	0.623	0.270	0.357
ADRB3	0.142	0.709	0.191	0.486	0.244	0.222
ADRA2B	0.169	0.497	0.177	0.519	0.204	0.358
ADRA2C	0.187	0.612	0.178	0.624	0.267	0.214
DRD5	0.154	0.755	0.186	0.621	0.280	0.270
CXCR1	0.136	0.608	0.205	0.348	0.279	-0.248
HTR1B	0.182	0.740	0.206	0.667	0.243	0.556
HTR2C	0.173	0.655	0.211	0.476	0.283	0.103
SSTR1	0.190	0.692	0.175	0.695	0.249	0.536
CCR1	0.192	0.141	0.169	0.402	0.235	0.045
DRD3	0.171	0.691	0.217	0.497	0.338	0.118
OPRK1	0.174	0.734	0.204	0.630	0.315	0.242
CCR5	0.255	0.110	0.233	0.223	0.394	-0.040

## Discussion

The results of the proposed approach suggest that the approach is capable of making valid activity predictions. First the Unified model showed that including structural target data is capable of producing a single model that is at least as good as separated QSAR modeling approaches, and secondly that it was capable of producing valid molecular inhibition inferences even for targets not present in the training set, albeit it is important that these have a given degree of structural similarity with at least one element in the training set.

In this study we actually covered two separate issues for ligand-target inhibition modelling: the first one was the proposed protein fingerprinting approach for identifying structural patterns, and the second one was the usage of Alphafold predicted structures, as these are readily available for most proteins known. Now the question is whether or not it would be possible to make similar inferences using current PDB structures, and whether the predictions would be of similar quality to the ones from Alphafold, or perhaps even better, as they do not come from simulated data. To achieve this goal, for our 144 targets we searched the availability of PDB structures, of which we found only 113 (approximately 78.5% ), not all of them complete. For many of the targets, more than one PDB structures were available, and the criterion selected was to use the structure and chain with the largest representation. Thus if one structure for a given protein captured only 87 residuals and another 395, the largest structure would be selected. This approach makes sense, so as to make the results directly comparable with the Alphafold based structures. Secondly we produced the target fingerprints using exactly the same procedure and parameters as described above (radius=5.0 Å and 16,381-bit fingerprints). Thirdly we recomputed the Principal Components for the new protein fingerprint data, as the dataset was smaller and slightly different from the original one and finally joined the projected protein data to the molecular activities.

For this comparison it was deemed sufficient to fit a new Unified Model as the differences from using PDB based data would emerge directly from it. Therefore two new model fitting procedures were executed, one with the PDB based data and another for the same targets and data, but using the Alphafold predicted structures. The data was partitioned into training and testing as before, using exactly the same partitions for both models. For model fitting we used the same procedure as above, using a Random Forest Regressor,increasing only the number of estimators to 200, as before. Results are very good overall but statistically indistinguishable (Table 4), suggesting that the approach indeed was able to capture structural characteristics from both sources of protein structure data. (The full source code for data processing as well as the resulting data sets is provided in the GitHub repository named below

**Table 4:** Result comparison of Unified Models, for the original data set and 113 targets with PDB data - RMSE - Root Mean Squared Errors of all predictions; RVE - Ratio of the Variance Explained.

Model	N. of targets	Training set size	Testing set size	RMSE	RVE
Original unified model	144	112271	28068	0.167	0.677
PDB structures	113	97592	24398	0.170	0.724
Alphafold structures	113	97592	24398	0.170	0.725

Processing raw PDB files from the PDB database is a complex process that requires several decisions for each individual protein. This is then when it becomes apparent the benefits of using Alphafold, even in the case where actual structures are known, as its usage was straightforward. The AlphaFold PDB files were easier to read and process, and finally, as results show, there was no benefit perceived in using the original experimentally determined data.

## Conclusions

This work had two essential goals. The first one A) had the purpose of showing that our approach of encoding protein structural information was capable of being used in a machine learning model that encompassed both target and ligand information. This was clearly achieved as the results of the Unified model unmistakably demonstrate, with results on par to individually fitted to QSAR models, and for goal B) we aimed to demonstrate that this modeling approach was capable of producing models for targets even if no binding information is available. The Semi Blind models also clearly suggested that this was the case. Even if the results were not homogeneous for all targets, and the quality of the predictions was not on par to the Unified models, there was again a significant result, which is very promising for tackling several existing problems in drug development.

Results from the AlphaFold Unified model were further compared with a new data set generated from experimentally determined protein structural data, and the results were actually statistically indistinguishable, suggesting that AlphaFold predicted structures were at least as good as crystallographically determined structures.

These results should be the inception for further research. Namely, to understand how to improve on the protein fingerprints collection and processing, as it is known that several amino acids show similar properties, and therefore several amino acid patterns should display similar behaviour, in similar structural conformations. Using such an enhanced representation could eventually produce a more comprehensive set of fingerprints, that would not require the usage of dimensionality reduction techniques. Other paths of research should include the usage of more sophisticated machine learning models, like deep learning models, that have provided excellent results with other proteo-chemometric approaches^29,30,47^. These models, however, would require much larger data sets, which, given the general availability of bioactivity data in public repositories, is a distinct possibility and should guarantee the feasibility of fitting very large models, possibly encompassing the full druggable human proteome, or even covering other species. The availability of structural data for virtually every protein in existence, as made available by AlphaFold, makes the development of such models simple and direct, unveiling their potential for application. Overall, these findings open up many possibilities for advancing research and understanding in the field, potentially providing a significant improvement in drug discovery and target prediction through improved machine learning approaches and comprehensive structural representations.

### Supplementary Information

Supplementary Information 1.

Supplementary Information 2.

## Data Availability

The data sets generated and/or analysed during the current study are available in the GitHub repository https://github.com/aofalcao/ProtLigModel The full source code for data processing and model fitting and testing is made available on GitHub in the above URL, and as supplementary material three different files are provided: SupMat 1 -The full results of the approaches for Baseline models, Unified Model and Semi-blind models. (3 CSV files in Zip format). SupMat 2 - Source code for Protein Fingerprint Generation (Python File) Requires a list of Uniprot IDs in a file and the set of AlphaFold predicted structures in PDB format
