# Author Correction: Inferring molecular inhibition potency with AlphaFold predicted structures

**DOI:** 10.1038/s41598-024-64154-w

**Published:** 2024-06-10

**Authors:** Pedro F. Oliveira, Rita C. Guedes, Andre O. Falcao

**Affiliations:** 1 Lasige, Faculdade de Ciências, Universidade de Lisboa, 1749-016 Lisboa, Portugal; 2 Research Institute for Medicines (iMed.ULisboa), Faculdade de Farmácia, Universidade de Lisboa, Av. Prof. Gama Pinto, 1649-003 Lisboa, Portugal; 3 Departamento de Informática, Faculdade de Ciências, Universidade de Lisboa, 1749-016 Lisboa, Portugal

Correction to: *Scientific Reports* 10.1038/s41598-024-58394-z, published online 08 April 2024

The original version of this Article contained an error in the Acknowledgements section.

Acknowledgements

“This was supported in part by Fundação para a Ciência e a Tecnologia through funding of project EXPL/QUI-OUT/1288/2021, titled: “ChemSlotProtein: Expanding the druggable universe to unlock chemists’ creativity”. The authors also acknowledge Fundação para a Ciência e a Tecnologia for its financial support UIDB/04138/2020 and UIDP/04138/2020 to iMed.Ulisboa. R.C.G. acknowledges funding from LISBOA-01-0246-FEDER-000017.”

Now reads

Acknowledgements

“This work was supported in part by Fundação para a Ciência e a Tecnologia through funding of project EXPL/QUI-OUT/1288/2021, titled: “ChemSlotProtein: Expanding the druggable universe to unlock chemists’ creativity”. The authors also acknowledge Fundação para a Ciência e a Tecnologia for its financial support UIDB/04138/2020 and UIDP/04138/2020 to iMed.Ulisboa. R.C.G. acknowledges funding from COMPETE LISBOA-01-0246-FEDER-000017 and by HEU Project Tclock4AD GA number 101072895, EXPL/QUI-OUT/1288/2021.”

The original Article has been corrected.

