# Supplementary material for: Inferring molecular inhibition potency with AlphaFold predicted structures
Source: Sci Rep. 2024 Apr 8;14:8252. doi: 10.1038/s41598-024-58394-z (PMC11001998; doi:10.1038/s41598-024-58394-z)
Supplement: Supplementary file 2 — Supplementary Information 2. [file 41598_2024_58394_MOESM2_ESM.pdf]

```python
import numpy as np
import pandas as pd
import scipy
import scipy.spatial
import pickle

def extractData(data):
    #bare bones PDB format reader (For AlphaFold retrieved structures)
    #returns a distance matrix between all atoms
    #        the coordinates of all atoms and
    #        the amino acids corresponding to each atom
    aas = {'ALA': 'A',
           'ARG': 'R',
           'ASN': 'N',
           'ASP': 'D',
           'ASX': 'B',
           'CYS': 'C',
           'GLU': 'E',
           'GLN': 'Q',
           'GLX': 'Z',
           'GLY': 'G',
           'HIS': 'H',
           'ILE': 'I',
           'LEU': 'L',
           'LYS': 'K',
           'MET': 'M',
           'PHE': 'F',
           'PRO': 'P',
           'SER': 'S',
           'THR': 'T',
           'TRP': 'W',
           'TYR': 'Y',
           'VAL': 'V'}
    #creates distance matrix, atom coordinates and atoms -> amino acids
    atoms_aas=[]
    coords=[]
    for line in data:
        ATOM = line[:4]
        if ATOM=="ATOM":
            #ATOM, aid, atm, aa, lixo, aaid, x,y,z, s1, s2, atm2=fs
            aid = line[6:11].strip()
            x = float(line[30:38].strip())
            y = float(line[38:46].strip())
            z = float(line[46:54].strip())
            aa = line[17:20].strip()
            aaid = line[22:26].strip()
            aid=int(aid)
            atoms_aas.append(aas[aa]+aaid)
            coords.append([x,y,z])

    coords=np.array(coords)
    atoms_aas=np.array(atoms_aas)
    DM = scipy.spatial.distance_matrix(coords, coords)
    return DM, coords, atoms_aas

def process_fp(fp):
    #returns processed aminoacids without ids from aas, but distinguishing different occurrences of the same AA
    L=sorted(list(fp))
    idx=0
    prev=L[0][0]
    L2=[prev+str(idx)]
    for i in L[1:]:
        if i[0]==prev: idx+=1
        else: idx=0
        prev=i[0]
        L2.append(prev+str(idx))
    return frozenset(L2)

def compute_fps(DM, coords, atoms_aas, max_dist=5):
    #computes the general structures for the fingerprints
    fps=[]
```

```python
    N=coords.shape[0]
    for i in range(N) :
        fp=set(atoms_aas[DM[i]<max_dist])
        if len(fp)>2:
            fps.append(process_fp(fp))
    return set(fps)

def getFPs(fname, max_dist=5):
    #reads a file, extracts the data and computes the structures for the fingerprints
    data=open(fname, "rt").readlines()
    dm, crds, aaas = extractData(data)
    fps=compute_fps(dm, crds, aaas, max_dist)

    return fps

def h(fp_set, n=16381):
    #hash and modulus function
    fp = [0]*n
    hs = [hash(h) % n for h in fp_set]
    hs = set(hs)
    for item in hs:
        fp[item] = 1
    return fp

# get protein ids retrieved from uniprot
ids = []


#this is where everything starts. Reads a list of uniprot ids from a file
with open(r'ids.txt', 'r') as fp:
    for line in fp:
        x = line[:-1]
        ids.append(x)


#for each id, open the corresponding PDB structure from AlphaFold,
# creates the hash maps (fingerprints)
# writes the fingerprints in Python's pickle format

for i in ids:
    dist = 5
    fname="pdb_files/{}.pdb".format(i)
    fps= getFPs(fname, dist)
    fps = h(fps)
    pickle.dump(fps, open("fp_dist{}_ki/{}_D{}.pickle".format(dist,i,dist), "wb"))
```
